# CmNAC25 targets *CmMYB6* to positively regulate anthocyanin biosynthesis during the post-flowering stage in chrysanthemum

**DOI:** 10.1186/s12915-023-01719-7

**Published:** 2023-10-09

**Authors:** Yuxi Wang, Yiguang Wang, Li-Jie Zhou, Jialin Peng, Chuwen Chen, Shenhui Liu, Aiping Song, Jiafu Jiang, Sumei Chen, Fadi Chen

**Affiliations:** State Key Laboratory of Crop Genetics and Germplasm Enhancement, Key Laboratory of Landscaping, Ministry of Agriculture and Rural Affairs, Key Laboratory of Biology of Ornamental Plants in East China, National Forestry and Grassland Administration, College of Horticulture, Nanjing Agricultural University, Nanjing, 210095 China

**Keywords:** Anthocyanin biosynthesis, Flower color, CmNAC25, MBW, *CmMYB6*, Post-flowering stage, Chrysanthemum

## Abstract

**Background:**

Anthocyanin is a class of important secondary metabolites that determines colorful petals in chrysanthemum, a famous cut flower. ‘Arctic Queen’ is a white chrysanthemum cultivar that does not accumulate anthocyanin during the flowering stage. During the post-flowering stage, the petals of ‘Arctic Queen’ accumulate anthocyanin and turn red. However, the molecular mechanism underlying this flower color change remains unclear.

**Results:**

In this study, by using transcriptome analysis, we identified *CmNAC25* as a candidate gene promoting anthocyanin accumulation in the post-flowering stage of ‘Arctic Queen’. CmNAC25 is directly bound to the promoter of *CmMYB6*, a core member of the MBW protein complex that promotes anthocyanin biosynthesis in chrysanthemum, to activate its expression. CmNAC25 also directly activates the promoter of *CmDFR*, which encodes the key enzyme in anthocyanin biosynthesis. *CmNAC25* was highly expressed during the post-flowering stage, while the expression level of *CmMYB#7*, a known R3 MYB transcription factor interfering with the formation of the CmMYB6–CmbHLH2 complex, significantly decreased. Genetic transformation of both chrysanthemum and *Nicotiana tabacum* verified that CmNAC25 was a positive regulator of anthocyanin biosynthesis. Another two cultivars that turned red during the post-flowering stages also demonstrated a similar mechanism.

**Conclusions:**

Altogether, our data revealed that CmNAC25 positively regulates anthocyanin biosynthesis in chrysanthemum petals during the post-flowering stages by directly activating *CmMYB6* and *CmDFR*. Our results thus revealed a crucial role of CmNAC25 in regulating flower color change during petal senescence and provided a target gene for molecular design breeding of flower color in chrysanthemum.

**Supplementary Information:**

The online version contains supplementary material available at 10.1186/s12915-023-01719-7.

## Background

Anthocyanins are secondary metabolites belonging to the class of flavonoids that confer a wide range of colors, including pink, orange, scarlet, purple, and blue, to higher plants [1–3]. Anthocyanins also play a role in multiple biological activities in plants, including attracting pollinators and seed dispersers and scavenging free radicals [4, 5].

The anthocyanin biosynthetic pathway is a significant branch of the general phenylpropanoid pathway [6]. The first committed step of flavonoid biosynthesis is catalyzed by chalcone synthase (CHS). CHS, known as the gatekeeper of flavonoid biosynthesis, plays a vital role in regulating the flavonoid biosynthesis pathway [7]. The enzymes involved in the biosynthesis of anthocyanins from 4-coumaroyl CoA are chalcone synthase (CHS), chalcone isomerase (CHI), flavanone 3-hydroxylase (F3H), dihydroflavonol reductase (DFR), anthocyanidin synthase (ANS, also called leucoanthocyanidin dioxygenase, LDOX), and UDP-glucose: flavonoid-3-O-glucosyltransferase (UFGT) [8].

Many factors regulate flower color. The color of anthocyanins changes due to pH, co-existing colorless compounds (co-pigments, typically flavones and flavonols), and metal ions [8]. Over the past several decades, research on the regulation of the anthocyanin biosynthetic pathway has increased [9, 10]. Studies have demonstrated that the expression of anthocyanin structural genes is regulated by the canonical MBW (MYB-bHLH-WD40) complex, which is conserved across different species [11]. MBW complex involved in anthocyanin biosynthesis has been characterized in many species; for example, TTG1-GL3/TT8-PAP1 likely forms the only MBW complex that activates the high production of anthocyanins in *pap1-D* in *Arabidopsis thaliana* [6]. In *Chrysanthemum* *morifolium*, MYB6-bHLH2 cooperates to promote the expression of anthocyanin-associated structural genes, including *CmDFR*, to affect the accumulation of anthocyanins [12].

Conversely, R3 MYB transcription factors can maintain their repressive function by directly inhibiting anthocyanin-associated structural genes or altering the MBW complex by competing with bHLH proteins for binding with R2R3 MYB TFs. In *Arabidopsis*, the R3 MYB transcription factor *AtCPC* suppresses anthocyanin biosynthesis by downregulating the expression of anthocyanin-associated structural genes [13]. R3 MYB-like transcription factor *PtrRML1* inhibits anthocyanin biosynthesis via a similar mechanism in poplar [14]. In white chrysanthemum cultivar ‘Jimba’, R3 MYB transcription factor CmMYB#7 inhibited anthocyanin biosynthesis by interacting with CmbHLH2 to destabilize the formation of CmMYB6-CmbHLH2 protein complex [15]. Subgroup 4 MYB transcription factors in *Arabidopsis thaliana*, MYB4, interact with the bHLH transcription factors, TT8, GL3, and EGL3, thereby interfering with the transcriptional activity of the MBW complexes [16].

Furthermore, the MBW complex is regulated by various upstream factors. In *Arabidopsis*, AtHY5 regulates *PAP1* expression via direct binding to G- and ACE-boxes in the promoter region, suggesting bifurcate regulation of anthocyanin biosynthesis by AtHY5 via transcriptional activation of *AtPAP1* [17]. MdMYB1 is sumoylated and stabilized at protein levels by SUMO E3 ligase MdSIZ1 at low temperatures [18]. In the existing floral patterning model in petunia, AN2 (MYB) determines intense petal limb pigmentation, and AN4 (MYB) controls flower tube and anther pigmentation, with standard basic helix–loop–helix (bHLH) (AN1) and WD-repeat (WDR) (AN11) partners [19]. An anthocyanin repressor, *MYB27*, not only directly limits the expression of anthocyanin biosynthesis structural genes but also represses the expression of the gene for the key bHLH factor of the MBW activation complex (*AN1*) [20]. Different regulatory factors are involved in regulating the MBW protein complex in plants. In addition, the regulatory mechanism of anthocyanin biosynthesis differs depending on the types of plants, such as edible or ornamental plants.

The flowering stage of ornamental plants begins with the bud stage and ends at the fully blooming stage. During the post-flowering stage, the anther dehiscence, the petals gradually senesce. For most ornamental plants, the anthocyanin accumulation decreases, and petal color fades during the post-flowering stage. For example, the flower color of *Paeonia* ‘Coral Sunset’ and ‘Pink Hawaiian Coral’ changes from pink to pale yellow at the post-flowering stage [21]. The flower color in *Malus* *hupehensis* (Pamp.) Rehder changes from red to white at the post-flowering stage [22]. In *Rosa* *hybrida*, the senescence of flowers results in color fading and loss of ornamental value [23]. However, for some chrysanthemum cultivars, anthocyanin does not accumulate in petals at the flowering stage but at the post-flowering stage, thereby turning petals red [24]. Such flower color changes may help eliminate excess reactive oxygen species in senescent petals and affect the ornamental quality of flowers. However, the detailed molecular mechanism underlying anthocyanin accumulation and flower color change during the post-flowering stage remains elusive.

Chrysanthemum, one of the world’s four prominent cut flowers, is a perennial flower of the genus *Chrysanthemum* *morifolium* belonging to the Compositae family. Anthocyanins in chrysanthemum are mainly cyanidin derivatives [25]. Chrysanthemum is widely used to study the regulation of anthocyanin biosynthesis owing to its colorful flowers and variable coloring patterns. Previous studies have reported several CmMYBs and CmbHLHs involved in regulating anthocyanin biosynthesis [12, 15, 26–28]. Among these regulatory factors, a complex formed by the CmMYB6-CmbHLH2 protein is crucial for activating anthocyanin accumulation in chrysanthemum flowers [12]. However, studies on potential regulators upstream of the complex in chrysanthemum are still scarce. Exploring the potential regulators upstream of the complex in chrysanthemum may provide insights into the regulatory mechanisms of anthocyanin biosynthesis in plants. We chose the white chrysanthemum cultivar ‘Arctic Queen’ for this study. Anthocyanin accumulation in the petals of ‘Arctic Queen’ does not occur at the flowering stage but at the post-flowering stage, gradually turning the petals red. Using transcriptome analysis, we identified a NAC transcription factor, CmNAC25, that is upregulated during the post-flowering stage of ‘Arctic Queen’. During the post-flowering stage, CmMYB#7, a known R3 MYB protein that interferes with the formation of the CmMYB6-CmbHLH2 protein complex [15], was significantly downregulated to facilitate the function of CmMYB6. CmNAC25 activated the expression of *CmMYB6* by directly binding to its promoter at the ACGT element located at − 89 ~  − 85 bp to initiate anthocyanin biosynthesis. Genetic transformation of both chrysanthemum and *Nicotiana tabacum* verified that CmNAC25 was a positive regulator of anthocyanin biosynthesis. Our results thus revealed a vital role of CmNAC25 in regulating flower color changes during the post-flowering stage in Chrysanthemum.

## Results

### The floral color of chrysanthemum ‘Arctic Queen’ turned red at post-flowering stages due to anthocyanin accumulation in petals

**Fig. 1:**
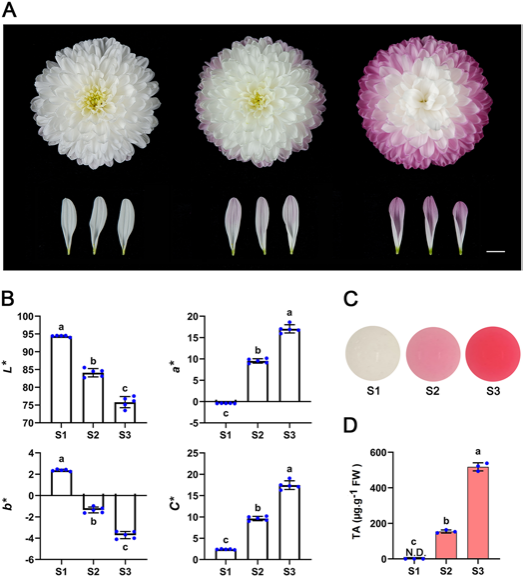
Anthocyanin accumulation in petals of chrysanthemum ‘Arctic Queen’ induced floral coloration at post-flowering stages. Bars = 1 cm. **A** Variation of floral color at the post-flowering stage. S1 (white), S2 (slightly red), S3 (obvious red). **B** CIEL*a*b* color parameters of petals at three flowering stages. Error bars indicate SD of five biological replicates. **C** Anthocyanin extraction from petals at three flowering stages. **D** The total anthocyanin content (TA) in petals at three flowering stages. Error bars indicate SD of three biological replicates. Different lowercase letters in **B** and **D** indicate significant differences (*p* < 0.05, ANOVA, Tukey’s correction)

‘Arctic Queen’, a cut-flower cultivar, was used in this study (Fig. 1). To analyze the color variation of chrysanthemum petals at the post-flowering stage, the flowering process of inflorescences after fully opened was divided into three stages, including S1 (fully blooming stage), S2 (10 days after full blooming), and S3 (20 days after full blooming) (Fig. 1A). As shown in Fig. 1A, petal color of outermost ray florets was white at S1 and changed to slight red at S2 and more reddish at S3. CIEL*a*b* analysis of petal color (Fig. 1B) showed that a* (redness) and C* (chroma) value of petals gradually increased, whereas L* (lightness) and b* (yellow-blue) values gradually decreased from S1 to S3. The quantification of pigments showed that no anthocyanins were detected in the petals at S1, and petals from S2 to S3 contained increased anthocyanins, which is consistent with the phenotype (Fig. 1C, D). These results suggest that the cultivar of the petals of ‘Arctic Queen’ gradually turned red in post-flowering stages due to the accumulation of anthocyanins.

### Transcriptomic profiling of chrysanthemum petals at three flowering stages

Total RNA extracted from the outermost ray florets of ‘Arctic Queen’ sampled at stages S1, S2, and S3 provided the template for RNA-Seq analysis. A total of 9 samples (three biological replicates for each of the three stages) were obtained. The outcome of the assembly procedure was a set of 139,504 unigene sequences with a mean length of 1138 bp, an N50 of 1773 bp, and a GC content of 39.09% (Additional file 1: Table S1).

**Fig. 2:**
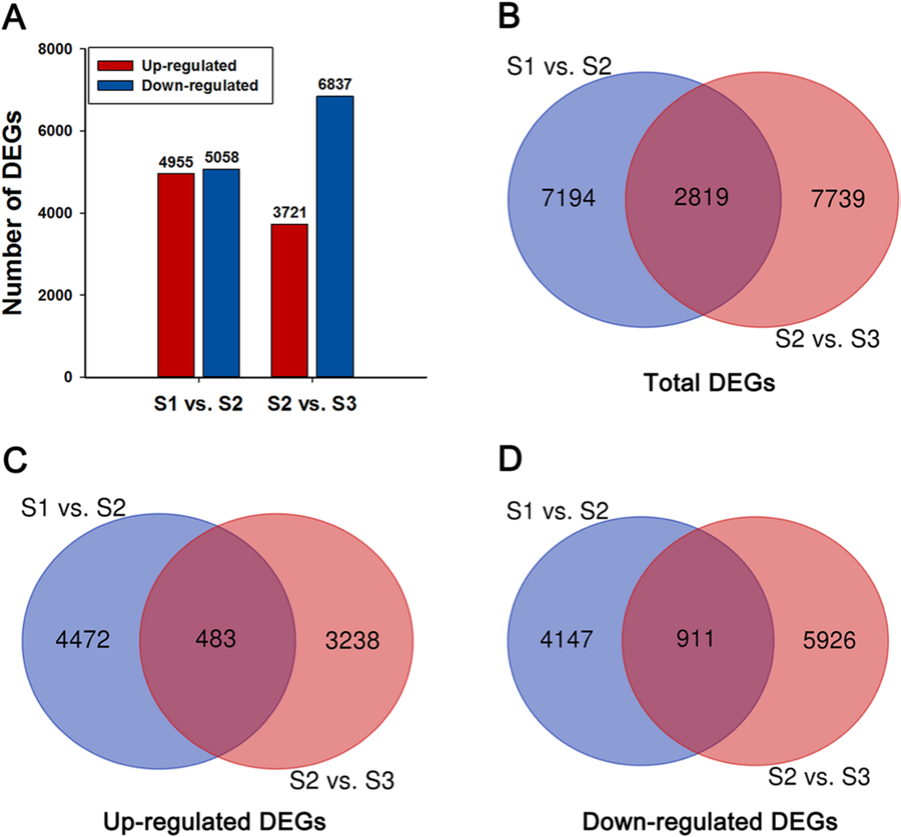
DEG analysis in the transcriptome of ‘Arctic Queen’ petals between different post-flowering stages. **A** The number of upregulated and downregulated DEGs in S1 vs. S2 and S2 vs. S3. **B** Venn diagrams of total DEGs in S1 vs S2 and S2 vs S3. **C**, **D** Venn diagrams of upregulated and downregulated DEGs in S1 vs S2 and S2 vs S3

Differential expression genes (DEGs) were analyzed by pairwise comparisons of transcriptomes between different flowering stages (S1, S2, and S3) (Fig. 2). There were 4955 genes expressed significantly higher in S2 than in S1, and 3721 genes higher expressed in S3 than in S2. However, there were 5058 genes expressed significantly lower in S2 than in S1 and 6837 genes lower expressed in S3 than in S2 (Fig. 2A). There were 2819 DEGs in both pairwise comparisons of S1 vs. S2 and S2 vs. S3 (Fig. 2B), of which 483 and 911 were simultaneously upregulated and downregulated DEGs, respectively (Fig. 2C, D).

### DEGs in the anthocyanin biosynthesis pathway

**Fig. 3:**
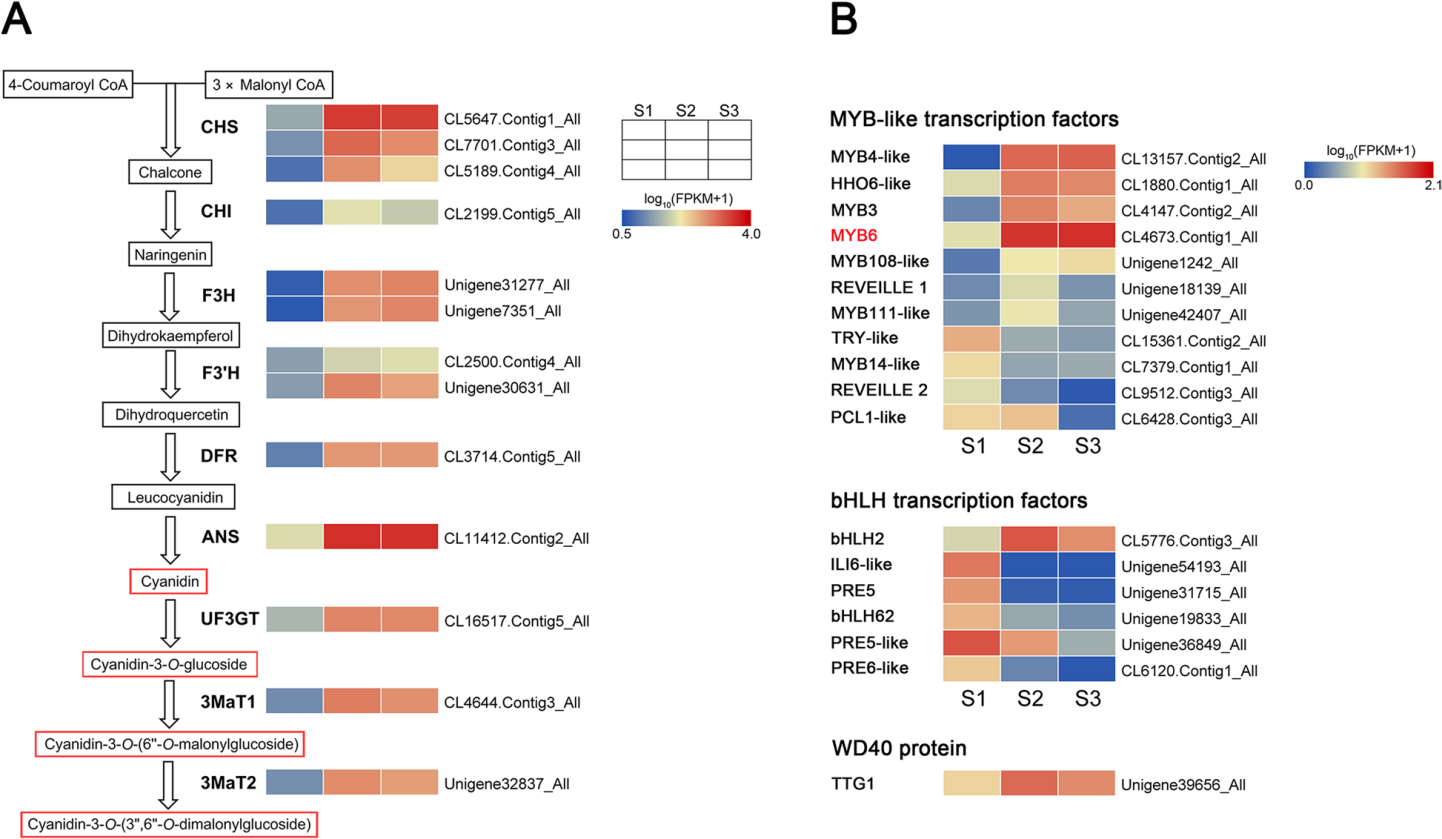
Differential expression of genes involved in anthocyanin biosynthesis pathway in petals of ‘Arctic Queen’ at three flowering stages. **A** Expression pattern of structural genes in the anthocyanin biosynthesis pathway of chrysanthemum. **B** Differential expression of genes encoding MYB-like, bHLH, and WD40 transcription factors. Heat maps depict normalized gene expression values (log_10_[FPKM + 1]), of which FPKM values represent the means of three biological replicates

To explore the molecular mechanism of anthocyanin accumulation leading to the coloration of ‘Arctic Queen’ petals at the post-flowering stages, the expression profiles of DEGs related to anthocyanin biosynthesis were further analyzed (Fig. 3). As shown in Fig. 3A, orthologs of structural genes in anthocyanin biosynthesis pathway, including *CHS*, *CHI*, *F3H*, *F3’H*, *DFR*, *UF3GT*, *3MaT1*, and *3MaT2*, were simultaneously upregulated from S1 to S2, correlating to the anthocyanin accumulation in petals (Fig. 1). This result indicates that potential upstream transcription factors may regulate multiple structural genes for anthocyanin accumulation transcriptionally.

Therefore, multiple DEGs encoding MYB-like, bHLH and WD40 transcription factors were identified. Figure 3B shows the expression profiles of the representative *MYBs*, *bHLHs*, and a *WD40*. Several orthologs of *MYBs*, including CL13157.Contig2_All, CL1880.Contig1_All, CL4147.Contig2_All, CL4673.Contig1_All, Unigene1242_All, Unigene18139_All, and Unigene42407_All, were upregulated from S1 to S2 (Fig. 3B). Among orthologs of *bHLHs* in Fig. 3B, only CL5776.Contig3_All was upregulated from S1 to S2, and an ortholog of *WD40* (Unigene39656_All) was upregulated from S1 to S2. Among these genes, *CmMYB6* (CL4673.Contig1_All homolog) and *CmbHLH2* (CL5776.Contig3_All homolog) reportedly promote anthocyanin biosynthesis via forming a complex in the chrysanthemum [12]. Furthermore, *CmMYB6* was upregulated with higher FPKM values than other regulatory genes (Fig. 3B), indicating its crucial role in the anthocyanin accumulation in senescent petals.

The anthocyanin biosynthesis is regulated by the classic MBW complex [11]. According to the above analysis of DEGs, we speculate that *CmMYB6* (CL4673.Contig1_All homolog), *CmbHLH2* (CL5776.Contig3_All homolog), and *CmTTG1* (Unigene39656_All homolog) were candidate members of the MBW complex regulating flower coloration at post-flowering stages. Therefore, the Y2H assay and BiFC assay were performed to detect the interactions among CmTTG1 and CmMYB6/CmbHLH2 referring to previous studies [20, 29]. As a result, CmTTG1 interacted with both CmMYB6 and CmbHLH2 (Additional file 4: Fig. S1A and B). As CmMYB6 and CmbHLH2 form a protein complex [12], this raised a question that whether CmTTG1 abrogates the interaction between CmMYB6 and CmbHLH2. To do this, an Y3H assay was performed as described by Schwenk et al. [30]. As demonstrated by Additional file 4: Fig. S1C, the yeast cells co-expressed with CmMYB6 and CmbHLH2 could grow on the selected medium but the combination of AD-T + BD-Lam could not. Meanwhile, the addition of CmTTG1 had no effect on the growth of yeast cells co-expressing CmMYB6 and CmbHLH2 on the selected medium (Additional file 4: Fig. S1C), indicating that CmTTG1 did not affect the interaction between CmMYB6 and CmbHLH2. Thus, we reason that CmMYB6, CmbHLH2, and CmTTG1 form a protein complex in chrysanthemum.

### Yeast one-hybrid screening and isolation of CmNAC25

Previous studies found that ‘Jimba’ occasionally and spontaneously produces red-colored petals under natural cultivation, namely ‘Turning red Jimba’, due to the change in expression of *CmMYB#7* [15]. In our research, we targeted the gradually turning red petals of ‘Arctic Queen’ at the post-flowering stage. The expression of *CmMYB#7* was also verified, and the result showed that it was also involved in the regulation of petal color change in white chrysanthemum cultivars at the post-flowering stage (Additional file 5: Fig. S2). The expression of *CmMYB#7* was high at the fully blooming stage (S1) and decreased significantly after entering the post-flowering stage (S2–S3) (Additional file 5: Fig. S2), which preliminarily shows why anthocyanins start to accumulate at post-flowering stages. However, the molecular mechanism of the gradual increase in the expression of a member of the MBW protein complex and anthocyanin accumulation at the post-flowering stage needs further exploration.

To explore the upstream regulatory mechanism of anthocyanin biosynthesis in chrysanthemum petals at post-flowering stages, a yeast one-hybrid (Y1H) screening using the promoter of *CmMYB6*, a core member of the chrysanthemum MBW complex, was carried out. The 1219-bp-long promoter of *CmMYB6* was isolated from genomic DNA of chrysanthemum and inserted into a pHIS2 vector as a bait to screen the cDNA library of ‘Arctic Queen’ petals, and several positive clones were identified by blast in transcriptome data (Additional file 2: Table S2). Among these genes, only a NAC transcription factor (Unigene39896_All) was upregulated from S1 to S2, correlating to the upregulation of *CmMYB6* and anthocyanin accumulation. Therefore, the full-length cDNA sequence of this gene, called *CmNAC25*, was cloned from ‘Arctic Queen’ for further identification. After amplification, ORFs of 921 bp were obtained for *CmNAC25*, encoding a protein consisting of 307 amino acids, with a predicted molecular weight of 33.77 kDa and an isoelectric point (pI) of 5.10.

**Fig. 4:**
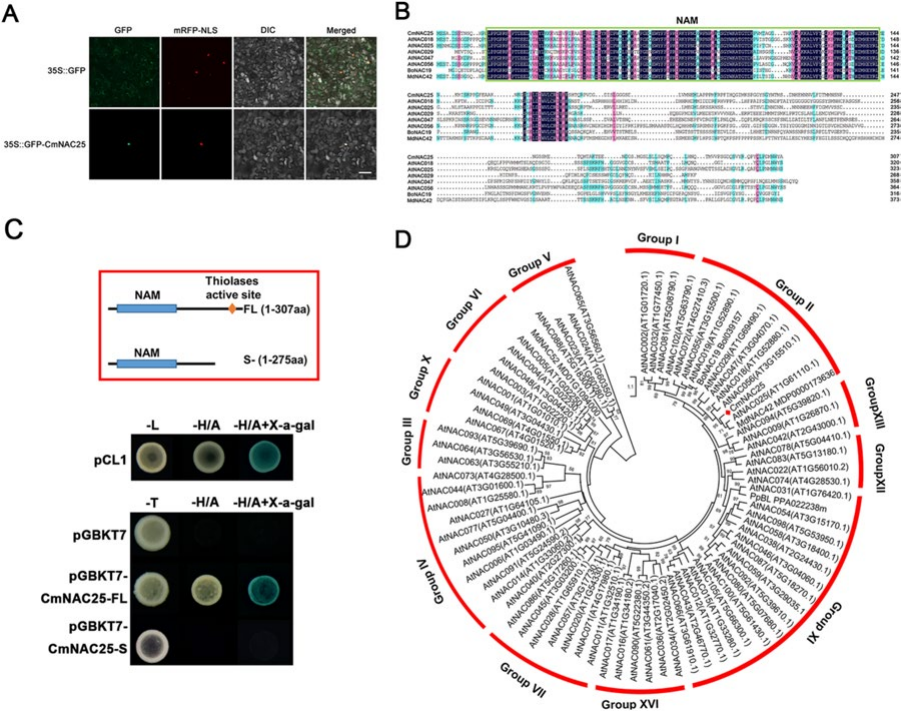
Subcellular localization, multiple sequence alignment, transcriptional activity, and phylogenetic analysis of CmNAC25. **A** Subcellular localization analysis of CmNAC25 in epidermal cells of *Nicotiana benthamiana* leaves. Bars = 50 μm. **B** Sequence alignment of CmNAC25 and its homologs from *Arabidopsis thaliana* (AtNAC018, AT1G52880; AtNAC025, AT1G61110; AtNAC029, AT1G60490; AtNAC047, AT3G04070; AtNAC056, AT3G15510), *Brassica* *oleracea* (BoNAC019, Bol039157), and *Malus* *domestica Borkh.* (MdNAC42, MDP0000173636). The green box indicates a conserved NAM domain. **C** Transcriptional activity analysis of CmNAC25 in a yeast system. The yeast cells transformed with the pCL1 construct were used as the positive control, and those with the empty vector pGBKT7 were used as the negative control. SD/-H/A indicates His and Ade’s synthetic dropout medium. **D** Phylogenetic analysis of NAC proteins from different species. Bar (1) indicates the branch length

For subcellular localization analysis of CmNAC25, 35S::GFP-CmNAC25, and 35S::D53-RFP constructs (nucleus markers) were transiently co-transformed into epidermal cells of *Nicotiana benthamiana* leaves. The co-transformation of the empty vector 35S::GFP and a 35S::D53-RFP construct was used as control. Figure 4A shows that the GFP signal of control was detected in both the cytoplasm and nucleus, while the GFP signal of 35S::GFP-CmNAC25 was only detected in the nucleus. The result indicates that CmNA25 was localized in the nucleus. Sequence alignment analysis shows that CmNAC25 harbors a conserved NAM domain in its N-terminus (Fig. 4B), which is a well-known specific domain of the NAC (NAM/ATAF/CUC) transcription factor family. Transcriptional activity analysis of CmNAC25 was performed in a yeast system, with the transformation of pCL1 as the positive control and the empty vector pGBKT7 as the negative control. The growth of yeast cells in SD/-H/A medium indicates that the full length of CmNAC25 (CmNAC25-FL, 1-307aa) has transcriptional activity. In contrast, truncated CmNAC25 (CmNAC25-S, 1-275aa) without the thiolase active site has no transcriptional activating activity (Fig. 4C). To analyze the phylogenetic relationships between CmNAC25 and NAC transcription factors in other plant species, a neighbor-joining phylogenetic tree was constructed. As shown in Fig. 4D, the NAC family was divided into several groups, similar to previous studies [31, 32]. Moreover, CmNAC25 was most closely related to AtNAC25, belonging to Group II (Fig. 4D). These results suggest that CmNAC25, belonging to the NAC family, is probably a transcriptional activator.

### CmNAC25 directly binds and activates the promoters of *CmMYB6* and *CmDFR*

**Fig. 5:**
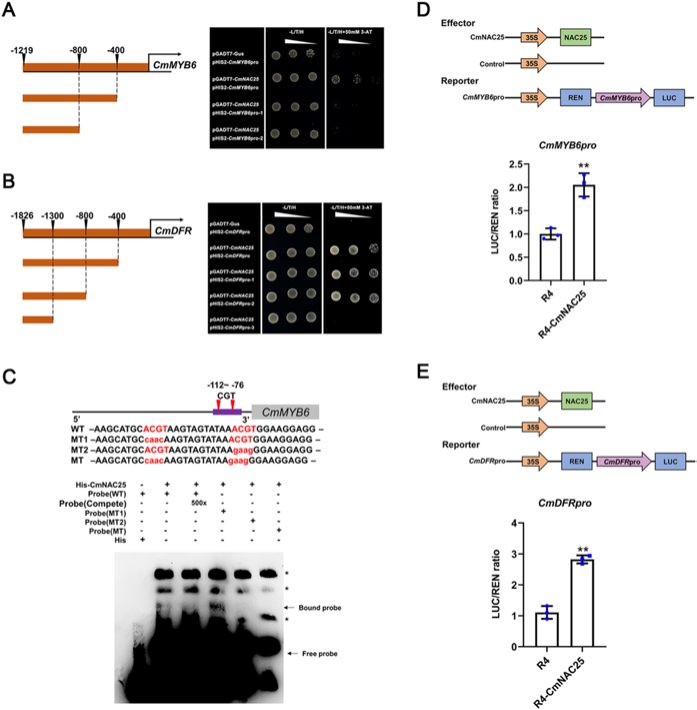
CmNAC25 activates the promoters of *CmMYB6* and *CmDFR*. **A**, **B** Interactions between CmNAC25 and different fragments of *CmMYB6* or *CmDFR* promoter in Y1H assays. Yeast cells co-transformed with pGADT7-Gus and pHIS2 constructs were used as negative controls. SD/-L/T/H indicates Leu, Trp, and His synthetic dropout medium. **C** EMSA assay showing that CmNAC25 directly binds to *CmMYB6* promoter at the ACGT element located at − 89 ~  − 85 bp. * means non-specific binding band. **D**, **E** Dual-luciferase assays showing that CmNAC25 activates the promoter activities of *CmMYB6* and *CmDFR*. LUC driven by the *CmMYB6* or *CmDFR* promoter was used as a reporter. The pORE-R4-35S::NAC25 construct was an effector, and the empty vector (pORE-R4) served as a control. The LUC/REN ratio of controls was set as 1. Error bars indicate the SD of three biological replicates. Samples denoted by asterisks indicate significant differences (*p* < 0.05, *t*-test)

To further explore the role of CmNAC25 in floral coloration, the interaction between CmNAC25 and the promoters of *CmMYB6* or the key structure genes was performed using Y1H assays, the results revealed that CmNAC25 can directly bind to the promoters of *CmMYB6* or the key structure gene *CmDFR* (Fig. 5A, B) rather than other structure genes (Additional file 6: Fig. S3A). As shown in Fig. 5A, yeast cells co-transformed with pGADT7-CmNAC25 construct and pHIS2-*CmMYB6*pro (− 1219 bp) grew on SD/-L/T/H medium with or without 50 mM 3-AT. However, the transformants with pHIS2 constructs inserted with *CmMYB6*pro-1 (− 1219 ~  − 400 bp) or *CmMYB6*pro-2 (− 1219 ~  − 800 bp) could not grow on SD/-L/T/H medium supplied with 50 mM 3-AT. The result indicates that CmNAC25 binds the *CmMYB6* promoter at the − 400 ~ 0 bp region. According to a previous study, DNA-binding motifs for NAC domain proteins with the CGT sequence is an essential core of the NAC-binding sequence [33], and its reverse complement is ACG. We found two ACGT elements in the promoter region of *CmMYB6* at the − 400 ~ 0 bp region. Next, the electrophoretic mobility shift assay (EMSA) showed that the bound probe of the first mutant ACGT element was still present. In contrast, the bound probe disappeared when the second or both ACGT elements were mutated, indicating that CmNAC25 directly binds the *CmMYB6* promoter at the second ACGT element located at − 89 ~  − 85 bp (Fig. 5C). Figure 5B shows that the yeast cells co-transformed with pGADT7-CmNAC25 construct and pHIS2-*CmDFR*pro (− 1829 bp), pHIS2-*CmDFR*pro-1 (− 1826 ~  − 400 bp), or pHIS2-*CmDFR*pro-2 (− 1826 ~  − 800 bp) grew on SD/-L/T/H medium with or without 50 mM 3-AT. The constructs of the transformants with pHIS2-CmDFRpro-3 (− 1826 ~  − 1300 bp) could not grow on an SD/-L/T/H medium supplied with 50 mM 3-AT. The result suggests that CmNAC25 binds the *CmDFR* promoter at the − 1300 ~  − 800 bp region, which also contains an ACGT element, and EMSA showed that CmNAC25 can directly bind the *CmDFR* promoter at the second ACGT element located at − 839 ~  − 835 bp (Additional file 7: Fig. S4). An MBW complex formed by the CmMYB6-CmbHLH2 protein complex is crucial for activating anthocyanin accumulation in chrysanthemum, we have known that CmNAC25 directly binds to the promoter of *CmMYB6* through the above results; furthermore, the results of Y2H and BiFC assays showed that CmNAC25 did not interact with CmMYB6 or CmbHLH2 (Additional file 6: Fig. S3B and C). Meanwhile, Y3H assay showed similar results when we replaced CmTTG1 with CmNAC25 (Additional file 4: Fig. S1C), the results further indicated that neither CmNAC25 nor CmTTG1 affects the interaction between CmMYB6 and CmbHLH2.

Dual-luciferase assays in chrysanthemum protoplasts were conducted to investigate further how CmNAC25 regulates the expression of *CmMYB6* or *CmDFR*. The LUC/REN ratio of *CmMYB*6pro::Luc or *CmDFR*pro::Luc co-expressed with 35S::CmNAC25 was significantly increased compared to those of the controls (Fig. 5D, E). These results suggest that CmNAC25 positively regulates anthocyanin biosynthesis by activating the expression of *CmMYB6* and *CmDFR*.

### The expression pattern of *CmNAC25* correlates to anthocyanin accumulation in senescent petals of different chrysanthemum cultivars

**Fig. 6:**
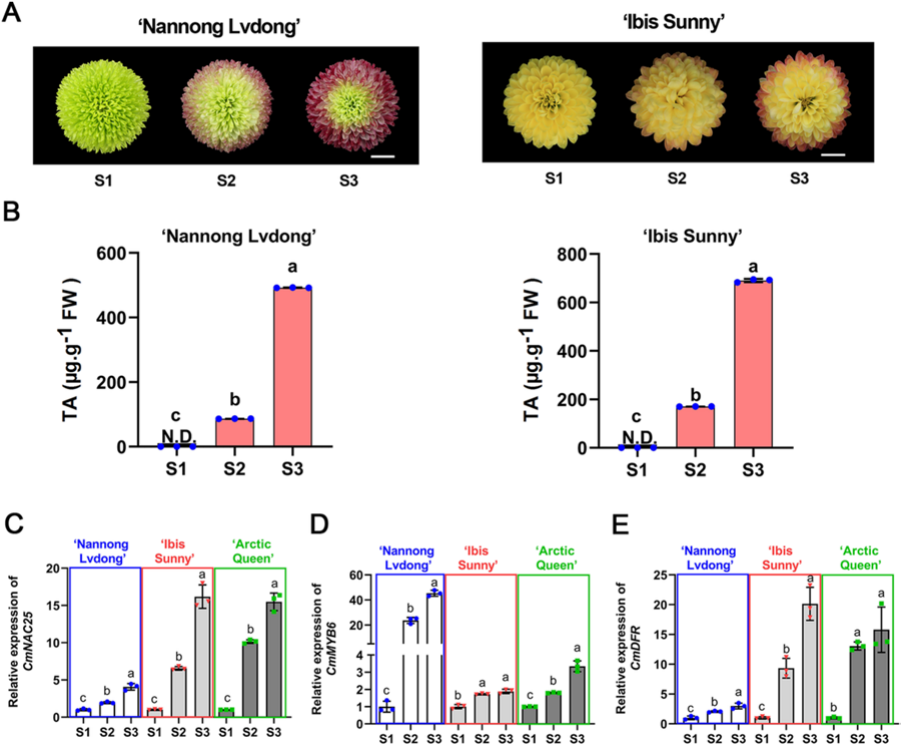
Anthocyanin content and expression patterns of *CmNAC25*, *CmMYB6*, and *CmDFR* in petals of different cultivars at the post-flowering stage. **A** The phenotype of ‘Nannong Lvdong’ and ‘Ibis Sunny’ petals at post-flowering stages. Bars = 1 cm. **B** The total anthocyanin content (TA) in petals of ‘Nannong Lvdong’ and ‘Ibis Sunny’ at three flowering stages. **C**–**E** Expression levels of *CmNAC25*, *CmMYB6*, and *CmDFR* in petals of ‘Nannong Lvdong’, ‘Ibis Sunny’, and ‘Arctic Queen’. Error bars indicate the SD of three biological replicates. Lowercase letters indicate significant differences (*p* < 0.05, ANOVA, Tukey’s correction)

To investigate whether *CmNAC25* regulates anthocyanin accumulation by promoting the expression of *CmMYB6* and *CmDFR* in different chrysanthemum cultivars, two more cultivars, ‘Nannong Lvdong’ and ‘Ibis Sunny’, were used for further research. Similar to ‘Arctic Queen’, the petals of these two cultivars also gradually turned red (Fig. 6A) with increased anthocyanins (Fig. 6B) at the post-flowering stages. The qRT-PCR analysis showed that the expression levels of *CmNAC25* were upregulated in petals of ‘Nannong Lvdong’, ‘Ibis Sunny’, and ‘Arctic Queen’ from S1 to S3 (Fig. 6C), consistent with the phenotypes. Moreover, the expression patterns of *CmMYB6* and *CmDFR* also had upregulated trend during the post-flowering process (Fig. 6D, E). These results further suggest that *CmNAC25*, a positive regulator of anthocyanin biosynthesis, has a similar molecular regulation mechanism in different chrysanthemum cultivars, which gradually turned red at the post-flowering stage.

### Overexpression of *CmNAC25* promotes anthocyanin accumulation in tobacco petals

**Fig. 7:**
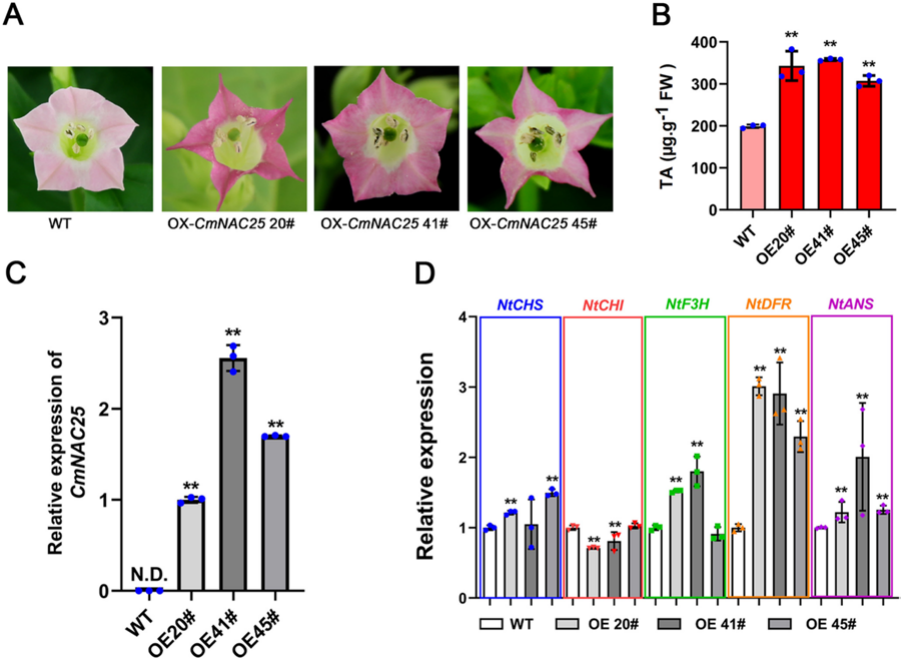
Overexpression of *CmNAC25* induced floral coloration and anthocyanin accumulation in tobacco. **A** The phenotypes of *CmNAC25*-overexpressing lines and WT plants. **B** Total anthocyanins (TA) in petals of WT and transgenic plants. **C** The transcripts of *CmNAC25* in transgenic lines and WT. N.D., No Detection. **D** The expression levels of *NtCHS*, *NtCHI*, *NtF3H*, *NtDFR*, and *NtANS* in petals of WT and transgenic plants. Error bars indicate SD of three biological replicates. Samples denoted by asterisks indicate significant differences (*p* < 0.05, *t*-test)

To confirm the function of *CmNAC25* in regulating anthocyanins biosynthesis, the pORE-R4-*CmNAC25* construct was transformed into tobacco (*Nicotiana tabacum*) using an agrobacterium-mediated method [34]. The T1 generation of four transgenic lines overexpressing *CmNAC25* were obtained and three of these (21#, 40#, and 45#) showed redder floral color than WT, while one of these showed similar floral color to WT (Fig. 7 and Additional file 8: Fig. S5). Compared to WT, three transgenic lines showed redder floral color (Fig. 7A) with increased anthocyanins in petals, the content of anthocyanins in transgenic lines 21#,40#, and 45# was about 72%, 80%, and 54% higher than that in WT, respectively (Fig. 7B). The qRT-PCR analysis indicated that *CmNAC25* transcripts were only expressed in the transgenic lines (Fig. 7C). Further, the expression level of *NtDFR* among endogenous genes was the most significantly induced in the transgenic lines (Fig. 7D). These results suggest that *CmNAC25* is a positive regulator of anthocyanin biosynthesis in plants.

### CmNAC25 regulates anthocyanin biosynthesis in chrysanthemum petals

**Fig. 8:**
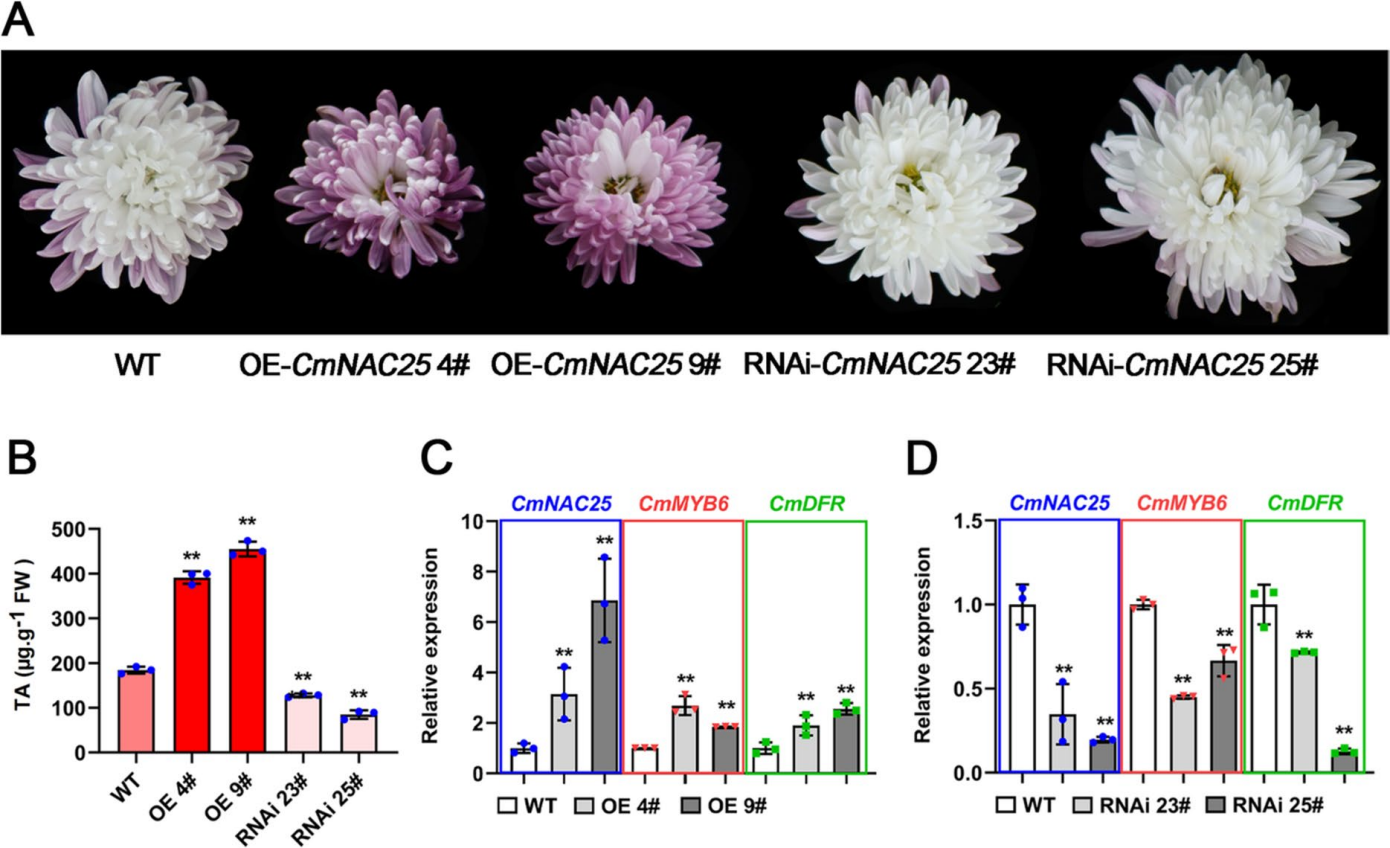
Overexpression and suppression of *CmNAC25* affected petal coloration and anthocyanin biosynthesis in the transgenic chrysanthemum ‘Jinba’. **A** The floral phenotypes of WT and transgenic lines OE-*CmNAC25* 4#, 9#, and RNAi-*CmNAC25* 23#, 25# at the initially senescent stage. **B** The total anthocyanin content (TA) in petals of WT and transgenic plants. **C** The expression of *CmNAC25*, *CmMYB6*, and *CmDFR* in OE-*CmNAC25* transgenic lines and WT. **D** The expression of *CmNAC25*, *CmMYB6*, and *CmDFR* in RNAi-*CmNAC25* transgenic lines and WT. Error bars indicate SD of three biological replicates. Samples denoted by asterisks indicate significant differences between WT and transgenic lines (*p* < 0.05, *t*-test)

To further characterize the function of *CmNAC25* in chrysanthemum, a chrysanthemum cultivar ‘Jinba’ with an available genetic transformation system was selected to obtain overexpressed-*CmNAC25* and RNAi-*CmNAC25* transgenic lines (Fig. 8A). As shown in Fig. 8A, compared with WT, the petals of two OE-*CmNAC25* lines 4# and 9# showed redder color. In comparison, the paler shade of petals was observed in two RNAi-*CmNAC25* lines, 23# and 25#, at the initially senescent stage (10 days after the fully blooming stage). Moreover, significantly more anthocyanin content was detected in petals of overexpressed-*CmNAC25* plants. In contrast, the petals of RNAi-*CmNAC25* transgenic lines contained fewer anthocyanins than the petals of WT, which was consistent with the phenotypes (Fig. 8B).

The qRT-PCR analysis showed that compared to WT, the expression levels of *CmMYB6* and *CmDFR*, being consistent with *CmNAC25* expression, were significantly increased in two overexpressed-*CmNAC25* lines (Fig. 8C). In contrast, the expression levels of these three genes were significantly downregulated in two RNAi-*CmNAC25* lines (Fig. 8D). Above results further suggest that CmNAC25 positively regulates anthocyanin biosynthesis by promoting *CmMYB6* and *CmDFR* expression in chrysanthemum petals at post-flowering stages. Furthermore, *CmNAC25-*mediated accumulation of anthocyanin in transgenic ‘Jinba’ only occurs at the post-flowering stage. The overexpressed-*CmNAC25* lines at the fully blooming stage are still white; this should be attributed to high expression of *CmMYB#7* disrupting the CmMYB6-CmbHLH2 protein complex at the flowering stage, disrupting anthocyanin biosynthesis [15].

Furthermore, to obtain more information for potential down-stream regulatory network of CmNAC25, petals of WT and RNAi-*CmNAC25* transgenic line 23# (RNAi23#) at the initially senescent stage were used for RNA-Seq analysis (Additional file 9: Fig. S6). There were 8120 DEGs detected in pairwise comparisons of transcriptomes between petals of WT and RNAi23#. Comparing to WT, there were 5149 DEGs upregulated and 2971 DEGs downregulated in RNAi23# (Additional file 9: Fig. S6A). In anthocyanin biosynthesis pathway, most structural genes including *CmDFR* were downregulated in RNAi23#, corresponding to fewer anthocyanins in RNAi23# petals than in WT (Additional file 9: Fig. S6B). Multiple genes encoding transcription factors like MYB6, MYB114, MYB111, and bHLH63 were downregulated, and EGL3 (bHLH) was upregulated in RNAi23# petals. However, there was no significant difference in expression levels of *MYB#7* between WT and RNAi23# (Additional file 9: Fig. S6C). These results suggested that in transgenic lines, CmNAC25 regulated anthocyanin biosynthesis via regulation on *CmMYB6*, *CmDFR* and maybe other genes, rather than *MYB#7*.

## Discussion

As a significant cut-flower material, the colors of chrysanthemum are of great economic value. Studies have shown that the pink, red, and purple petals of chrysanthemum are provided by different contents of anthocyanins [35]. In some chrysanthemum cultivars, petal color changes during flowering, including deepening color and fading of petal color, caused by changes in anthocyanin content, which greatly impact the economic value of chrysanthemums. Understanding the molecular mechanism of the above change process is very important for the molecular breeding of chrysanthemum.

In the process of petal senescence, there will be changes in a series of physiological indicators, as well as changes in petal color. For some ornamental plants, the anther dehiscence, and the petal color fades; for example, the flower color of *Paeonia* ‘Coral Sunset’ and ‘Pink Hawaiian Coral’ changes from pink to pale yellow at post-flowering stages [21], and the flowers change from red to white during development in *Malus* *hupehensis* (Pamp.) Rehder [22], in *Rosa* *hybrida*, flower senescence with fading color and loss of ornamental value [23]. When chrysanthemum petals enter the senescence stage after blooming, the color changes that occur are divided into two categories [24]. One is that the content of anthocyanin decreases and the color of the petals fades; anthocyanin biosynthesis is an energy-consuming process because the redox processes involved require high energy [36], reducing its biosynthesis at post-flowering may be to save energy and prolong the flowering period; while the other is that anthocyanins begin to accumulate and the flower color gradually turns red, such as the plant material selected in this study, i.e., ‘Arctic Queen’ ‘Nannong Lvdong’, and ‘Ibis Sunny’. We found that the gradual accumulation of anthocyanins caused the above phenotype. Anther dehiscence determines the available pollen foragers can collect, so pollination by insects such as bees occurs after the anthers dehiscence [37]. This means that anthocyanins begin to accumulate at the post-flowering stage, and petals gradually turn red may be significant for attracting insect pollinators. In addition, plants produce a large number of reactive oxygen species (ROS) when it senescence [38], and it is known that anthocyanins can scavenge free radicals [4, 5], so the accumulation of anthocyanins at the post-flowering stage may also participate in the scavenging ROS in petals.

Anthocyanin is a product of the flavonoid biosynthetic pathway, produced under the continuous catalysis of a series of structural genes in this pathway [8]. Previous studies have shown that this pathway is positively regulated by the canonical MBW complex [11], which was characterized as MYB6-bHLH2 in chrysanthemum [12]. MYB TFs (transcription factors) are critical modulators of the structural genes in the flavonoid biosynthetic pathways [39]. Transcriptome analysis of ‘Arctic Queen’ samples at different stages showed that the expression level of structural genes related to the anthocyanin biosynthesis and the members of the MBW complex is consistent with increased anthocyanin accumulation (Figs. 1 and 3). It suggested that further upstream potential transcription factors may play a role via the MBW complex and a series of structural genes. Previous studies have shown that TFs act through the MBW complex to regulate anthocyanin biosynthesis; for example, HAT1 interacts with MYB75 and interferes with the MBW protein complex to suppress abundant anthocyanin phenotype of *pap1-D* plant in Arabidopsis [40]. Furthermore, PpMYB18, a negative regulator of anthocyanin and PA accumulation, can compete with MYB activators for binding to bHLHs to interfere with the MBW protein complex [41]. However, until now, TFs acting through the MBW complex have hardly been characterized in chrysanthemums.

We characterized the transcription factor upstream of *CmMYB6*, namely CmNAC25, it can act as a positive regulator of anthocyanin biosynthesis through promoting the expression of *CmMYB6* and *CmDFR*. Furthermore, transcriptome analysis of transgenic ‘Jinba’ and WT were performed. In RNAi-*CmNAC25* transgenic line, the expression of *CmNAC25* was significantly lower than that in WT, and the expression levels of not only *CmMYB6* and *CmDFR*, but also other genes of anthocyanin biosynthesis, were significantly reduced (Additional file 9: Fig. S6), because CmMYB6-CmbHLH2 was the upstream positive regulator of anthocyanin biosynthesis pathway [12, 42]. Meanwhile, we found some candidate differentially expressed TFs in RNAi-*CmNAC25* transgenic line compared with WT, such as several MYB TFs and bHLH TFs (Additional file 9: Fig. S6). It suggests that although CmNAC25 does not bind to promoters of other structrual genes, it may affect expression of these genes by regulating those potential transcription factors.

NAC (NAM, ATAF1/2, and CUC2) proteins are one of the most prominent plant-specific TF families with a well-conserved N-terminal NAM domain [43]. The NAC TFs have been shown to regulate several biological processes, including shoot apical meristem formation and maintenance, floral development, control of flowering induction in response to stresses, embryo development, hormone signaling, and regulation of secondary cell wall synthesis [32]. CmNAC25 belongs to the NAC gene family with a conserved NAM domain in its N-terminal (Fig. 4B). Previous studies suggested the transcriptional activation domain exists in the C-terminal region rather than the NAM domain [44, 45]. Our results also illustrated the same fact that the CmNAC25 sequence after truncating the C-terminus (CmNAC25-S) has no transcriptional activation activity (Fig. 4C). Phylogenetic analysis showed that NAC TF families are enormous and contain several subgroups as shown in Fig. 4D [31, 32], of which CmNAC25 belongs to Group II and is most closely related to AtNAC25. There are two other genes, BoNAC19 and MdNAC42, which are characterized as regulators of anthocyanin biosynthesis, and are also in Group II, they are closely related to CmNAC25. In *Malus* *domestica Borkh.*, MdNAC42 is an essential positive regulator of the regulatory network controlling the anthocyanin pigmentation of red-fleshed apples [46]. However, overexpression of *BoNAC019* reduces anthocyanin accumulation by decreasing expression levels of anthocyanin genes in Arabidopsis [47]. This suggests that even though in the same group of phylogenetic trees, the functions of *NAC* genes as regulators of anthocyanin biosynthesis have diverged. In our study, *CmNAC25* was characterized as a positive regulator of anthocyanin biosynthesis.

**Fig. 9:**
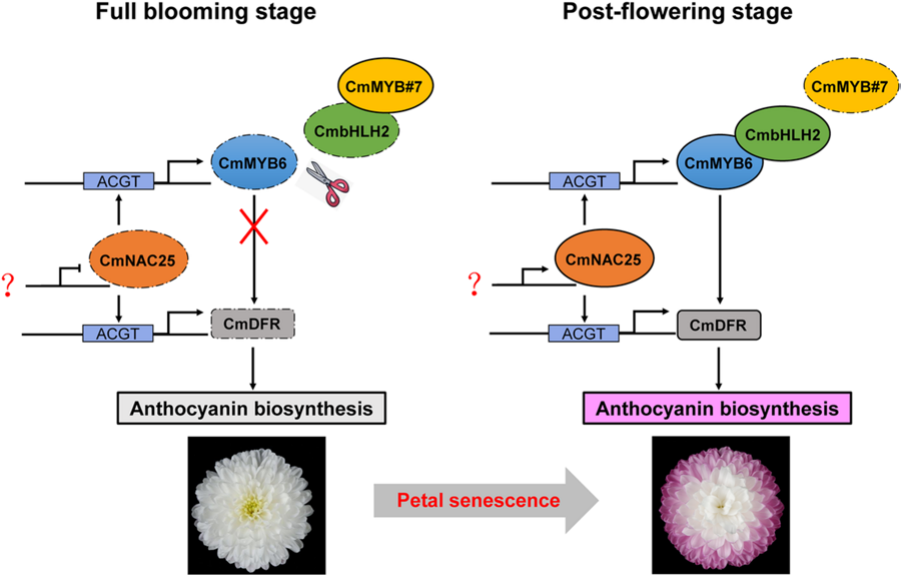
Proposed model for CmNAC25-regulated anthocyanin biosynthesis at post-flowering stages. Dashed boxes indicate that relative lower expression levels of *CmNAC25*, *CmMYB6*, *CmbHLH2*, and *CmDFR* at full blooming stage, and relative lower expression of *CmMYB#7* at post-flowering stage. Solid boxes indicate that *CmMYB#7* was highly expressed at full blooming stage, while the expression levels of *CmNAC25*, *CmMYB6*, *CmbHLH2*, and *CmDFR* were upregulated at post-flowering stage

In previous studies, Xiang et al. found that the reason for white appearance of ‘Jinba’ at the blooming stage was that the R3 MYB transcription factor CmMYB#7, which can interact with CmbHLH2, disrupted the MBW protein complex: CmMYB6-CmbHLH2, which is necessary for the anthocyanin biosynthesis of chrysanthemum, thus blocking the anthocyanin biosynthesis in white petals [15]. At the post-flowering stage, the expression of *CmMYB#7* is decreased, and the CmbHLH2 was released to form CmMYB6-CmbHLH2 complex, the petals began to accumulate anthocyanins [15]. It revealed the molecular mechanism of CmMYB#7 that negatively regulates the formation of MBW complex on protein level in chrysanthemum. In our study, we also found that in ‘Arctic Queen’, the start of anthocyanin accumulation may also be caused by the decreased expression of *CmMYB#7* (Additional file 5: Fig. S2) and the release of CmbHLH2 member of the MBW protein complex (Fig. 9). Further, we characterized the positive regulator CmNAC25 upstream of *CmMYB6* for the first time, and we revealed the regulation of transcriptional level during the gradual accumulation of anthocyanin in petals at the post-flowering stage in chrysanthemums. In RNAi-*CmNAC25* transgenic line, *CmMYB#7* was not diffentially expressed comparing to WT (Additional file 9: Fig. S6), suggestting that CmNAC25 was probabely not the upstream of *CmMYB#7*. Therefore, we elucidated that activator CmNAC25 and repressor CmMYB#7 play different roles in this coordinated regulation on anthocyanin accumulation which induces chrysanthemum floral color turning red at the post-flowering stage (Fig. 9).

## Conclusions

Overall, the molecular mechanisms of petal coloration in chrysanthemum during post-flowering periods were investigated. The gradual accumulation of anthocyanins at the post-flowering stage is manifested by the phenotype of gradually turning redder petals. A transcription factor, CmNAC25 which was upregulated in post-flowering petals, was confirmed to be upstream of *CmMYB6*, the member of the MBW complex, and key anthocyanin biosynthetic gene *CmDFR*. Moreover, it acts as a positive regulator of anthocyanin biosynthesis by directly or indirectly regulating multiple genes, which is approved by the phenotypes of transgenic plants (including tobacco and chrysanthemum). In conclusion, CmNAC25 plays a crucial role in anthocyanin-induced petal coloration of post-flowering chrysanthemum.

## Methods

### Plant materials

The chrysanthemum cultivars ‘Arctic Queen’, ‘Nannong Lvdong’, ‘Ibis Sunny’, and ‘Jinba’ used in this study were obtained from the Chrysanthemum Germplasm Resource Preserving Center (Nanjing Agricultural University, China). These chrysanthemum cultivars were cultivated in a greenhouse under short-day conditions (8 h/16 h light/dark, 24 °C/18 °C) with a relative humidity of 70%. The outer ray florets at three flowering stages (S1, S2, and S3) were collected from at least five individual plants of each cultivar for floral color measurement, and another portion of each sample was stored at − 80 °C for further analysis. The early-flowering tobacco cultivar ‘Xinashi’ (*Nicotiana tabacum*) described by Ning et al. [48] and chrysanthemum ‘Jinba’ were used for genetic transformation. The transgenetic tobacco and WT plants were cultivated in a greenhouse under long-day conditions (16 h/8 h light/dark, 25 °C/18 °C) with a relative humidity of 70%.

### Floral color measurement

The CIEL*a*b* parameters of the chrysanthemum’s floral color were measured using a Minolta CR-400 portable colorimeter (Konica Minolta, Japan). Values of lightness (L*) and the two chromatic components a* and b* were obtained from the measurements. According to the equation: C* = (a*^2^ + b*^2^)^1/2^, the values of C* were calculated [49]. Each sample was performed with five biological replicates.

### Determination of anthocyanins

The anthocyanins in petals of three chrysanthemum cultivars (‘Arctic Queen’, ‘Nannong Lvdong’, and ‘Ibis Sunny’) and transgenic plants (tobacco and ‘Jinba’) were extracted by using a solution of methanol/distilled water/formic acid/trifluoroacetic acid (70/27/2/1, v/v/v/v) at 4 °C in the dark for 24 h, following a previously described method [25]. All extracts were filtered through 0.22 μm and then used for HPLC analysis, which was performed using a Shimadzu HPLC system (Kyoto, Japan) equipped with an LC-20AD pump, an SPD-M20A DAD detector, a CTO-20A column oven, a SIL-20A auto-injector, and a C18 column of Inert Sustain (4.6 × 250 mm, 3 μm, Shimadzu GL, Shanghai, China). Anthocyanin contents were measured according to the method described in our previous study [27]. Each sample was performed with three biological replicates.

### Sample preparation, transcriptome sequencing, and differential expression genes (DEGs) analysis

The total RNA was extracted from petals of ‘Arctic Queen’ at S1, S2, and S3, and petals of ‘Jinba’ WT and RNAi-*CmNAC25* transgenic line 23# with RNAiso Reagent (Takara, Tokyo, Japan) according to the manufacturer’s instructions. Three biological replicates were conducted for each sample. Nine cDNA libraries for ‘Arctic Queen’, and six cDNA libraries for ‘Jinba’ WT and RNAi-*CmNAC25* transgenic line 23#, were constructed using these RNA samples and then sequenced using the Illumina HiSeq2500 platform at Beijing Genomics Institute (Shenzhen, China). The raw data obtained by sequencing is filtered to get clean reads. The clean reads were de novo assembled into transcripts using Trinity software [50], and the assembled transcripts were clustered and de-redundant using Tgicl software [51] to obtain unigenes. The assembled unigenes were annotated with NT, NR, COG, KEGG, and SwissProt using Blast [52]. GO annotation was performed using Blast2GO [53] and NR annotation results. InterProScan5 [54] was used for InterPro annotation. The clean reads were aligned to unigenes using Bowtie2 [55], and then RSEM [56] was used to calculate the gene expression levels of each sample, expressed as FPKM. Using the PossionDis [57] method, the DEGs between samples were detected according to the Poisson distribution principle, and the parameters were set as log2 (Fold Change) ≥ 1.00 and FDR ≤ 0.001. According to DEGs between different samples, Venn diagrams were drawn using the online website Venny 2.1 at https://bioinfogp.cnb.csic.es/tools/venny/index.html. Heat maps for DEGs in anthocyanin biosynthesis pathway were drawn with the normalized gene expression values (log_10_[FPKM + 1]) by using TBtools.

### Y1H assay

The cDNA library was constructed with total RNA extracted from ‘Arctic Queen’ petals at the S3 stage by Invitrogen company (Invitrogen, Shanghai, China). The promoter fragment of *CmMYB6* was cloned and inserted into a pHIS2 vector as a bait plasmid. To perform Y1H screening, the cDNA library and the bait plasmid were co-transformed into Y187 yeast (*Saccharomyces cerevisiae*) using the lithium acetate method. The yeast cells were selected on a selective medium lacking Trp, Leu, and His (SD/-Trp/-Leu/-His) supplemented with 50 mM 3AT at 28 °C for 3 days. The positive colonies were sequenced and blasted in the transcriptome database of ‘Arctic Queen’ petals.

For Y1H assays, the full-length coding sequence (CDS) of *CmNAC25*was inserted into the pGADT7 vector. The CDS of GUS (β-glucuronidase) was inserted into the pGADT7 vector as a negative control. Next, the promoter fragments of the corresponding genes were cloned into the pHIS2 vector. The primer pairs used for gene cloning are listed in Supplementary Additional file 3: Table S3. Subsequently, the pGADT7 and the pHIS2 construct were co-transformed into Y187 yeast using the lithium acetate method. Later, yeast cells were inoculated on SD/-Trp/-Leu/-His. The selected colonies were then inoculated on a -Trp/-Leu/-His medium supplemented with 50 mM 3-AT and grown for 3 days at 28 °C.

### Cloning and phylogenetic analysis of full-length cDNA of CmNAC25

First-strand cDNA was synthesized with total RNA of ‘Arctic Queen’ petals using M-MLV reverse transcriptase (TaKaRa, Tokyo, Japan), according to the manufacturer’s protocol. The full-length sequence of the *CmNAC25* open reading frame (ORF), which was obtained from the chrysanthemum transcriptome database, was cloned from the cDNA and inserted into the PMD19-T vector for further analysis. The homologs of CmNAC25 were obtained from NCBI (https://blast.ncbi.nlm.nih.gov/Blast.cgi) and TAIR (http://www.arabidopsis.org/) databases. Polypeptide alignment of CmNAC25 and its homologs from other species was performed using DNAMAN V6 software, and structural analysis of the CmNAC25 amino acid sequence was conducted using the ExPASy tool (https://prosite.expasy.org/). The amino acid sequences of CmNAC25 and its homologs from other species were used to construct phylogenetic trees based on the neighbor-joining method in MEGA 7.0 software, setting up bootstrap to test 1000 repetition [58].

### Subcellular localization assay

Subcellular localization assay was conducted using the method described in a previous study [59]. Briefly, the *CmNAC25* ORF was inserted into a pORE-R4 vector, a green fluorescent protein (GFP) fusion construct driven by the cauliflower mosaic virus promoter (CaMV 35S). Then, the 35S::*CmNAC25*-GFP construct and the 35S::GFP empty vector were transiently expressed in the epidermal cells of *Nicotiana benthamiana* leaves. After holding at 22 °C for 24–30 h, the samples were monitored for GFP activity using a Zeiss LSM 780 confocal microscope (Zeiss, Jena, Germany). The co-expressed 35S::D53-RFP construct was used as a nuclear marker.

### Transcriptional activity assay and Y2H assay

The transcriptional activity analysis of CmNAC25 was performed in a yeast system. The full-length CDS and an N-terminal fragment (1–275aa) of CmNAC25 ORF were inserted into a pGBKT7 vector. The pCL1 plasmid was used as a positive control, whereas the empty pGBKT7 vector was used as a negative control. The resultant vectors were then transformed into yeast strain Y2H (*Saccharomyces cerevisiae*) using the lithium acetate method. Subsequently, yeast cells containing plasmids of corresponding genes or negative control were inoculated on a selective medium lacking Trp, and yeast cells containing pCL1 plasmid were inoculated on a selective medium lacking, Leu. The selected colonies were then inoculated on a selective medium lacking His and Adenine (-H/A) with 0 or 20 mg/mL x-*α*-gal for 2 days at 28 °C. For Y2H assay, full-length CDSs of *CmTTG1*, *CmMYB6*, and *CmbHLH2* were inserted into the *pGADT7* vector as preys. And domain-deletion constructs of *CmMYB6* [N-(1-124aa)], *CmbHLH2* [C-(307-611aa)], and CmNAC25[S-(1-275aa)] were inserted into the *pGBKT7* vector as baits. The resultant preys and baits were then cotransformed into Y2H yeast (*Saccharomyces cerevisiae*) using the lithium acetate method. After inoculating on a selective medium lacking Trp and Leu (-T/-L), the positive colonies were inoculated on a selective medium lacking Trp, Leu, His, and adenine (-L/-T/-H/-A) and grown for 3 days at 28 °C to identify possible interactions.

### BiFC assay

For BiFC assays, the CDSs of *CmMYB6*, *CmbHLH2*, and *CmNAC25* were cloned into the *pSPYNE* vector, while the CDSs of *CmMYB6*, *CmbHLH2*, and *CmTTG1* were cloned into the *pSPYCE* vector [60]. The resultant vectors were introduced into *Agrobacterium* *tumefaciens* strain EHA105, and then transiently co-infiltrated in *N.* *benthamiana* leaves and visualized by fluorescence microscopy. The co-expressed *35S:D53-RFP* construct was used as a nuclear marker.

### Dual-luciferase assays

For transient expression assays, the promoter fragments of downstream genes were cloned into the pGreenII 0800-LUC vector using the primers listed in Additional file 3: Table S3 to generate reporter constructs. The pORE-R4-*CmNAC25* construct was used as an effector, and the empty pORE-R4 vector was used as a negative control. Subsequently, the resultant vectors were transiently expressed in chrysanthemum protoplast, referring to the method described by a previous study [61]. The LUC-to-REN activity ratio was measured using the Infinite M200 luminometer (Tecan, Mannerdorf, Switzerland) with the Dual-Glo® 694Luciferase Assay System (Promega, Beijing, China).

### Transformation of tobacco and chrysanthemum

The pORE-R4-*CmNAC25* construct was used for overexpressing *CmNAC25* in tobacco and chrysanthemum. To construct an amiRNAi vector, RNAi-*CmNAC25*-1/2/3/4 sequences were designed using the WMD3-Web app for the automated design of artificial microRNAs (http://wmd3.weigelworld.org/). The pBSK-miR319a plasmid was used as a template to amplify the fragment into binary vector pORE-R4, controlled by the 2×CaMV 35 S promoter. The transformation constructs pORE-R4-*CmNAC25* and RNAi-*CmNAC25* were introduced into *Agrobacterium* *tumefaciens* strain EHA105 cells by electro-transformation (1.8 kV, 5 s) [34].

Agrobacterium-mediated transformation of tobacco was performed following the method described in a previous study [48]. T1 plants of transgenic tobacco lines (WT tobacco was used as a control) were used for molecular and phenotypic analysis. The agrobacterium-mediated transformation of chrysanthemum ‘Jinba’ was performed as Wang et al. described [62]. Genomic DNA of OE-*CmNAC25* or RNAi-*CmNAC25* transgenic lines was isolated to confirm the presence of transgenes by PCR using the primer pairs of *CmNAC25*-ORF-R and 35 S-F or primer pairs of RNAi-*CmNAC25*-2 and 35 S-F, respectively (Additional file 3: Table S3). The petals of T1-generation transgenic tobacco lines, transgenic chrysanthemum lines, and corresponding WT plants were used for anthocyanin determination and qRT-PCR gene expression analysis. The petals of transgenic chrysanthemum line (RNAi23#) and corresponding WT plants at the initially senescent stage were used for RNA-Seq analysis.

### Quantitative real-time PCR analysis

Total RNA was isolated from the petals of three chrysanthemum cultivars (‘Arctic Queen’, ‘Nannong Lvdong’, and ‘Ibis Sunny’) at three flowering stages and transgenic plants (tobacco and ‘Jinba’) and used for synthesizing the first-strand cDNA. In chrysanthemum, *CmEF1α* (elongation factor-1 α) is one of the most stable housekeeping genes [63], while in tobacco, *NtEF1α* demonstrated the highest expression stability in a diverse set of 22 tobacco cDNA samples derived from developmentally distinct tissues and from plants exposed to several abiotic stresses [64]. So transcript abundance was assessed using SYBR Premix Ex Taq (TaKaRa, Tokyo, Japan) and LightCycler® 96 (Roche, Switzerland) real-time PCR system, with *CmEF1α* [63] and *NtEF1α* [65] as internal controls, respectively. The primers used for each target gene, some of which were obtained from the literature [66, 67], are listed in Additional file 3: Table S3. The relative expression levels of related genes were normalized to the relative expression level of *CmEF1α* or *NtEF1α*, using the 2^−△△Ct^ method [68].

### Supplementary Information

**Additional file 1: Table S1.** Summary of transcriptome sequencing data for ‘Arctic Queen’ petals at three flowering stages.

**Additional file 2: Table S2.** The predicted upstream regulators of the *CmMYB6* promoter identified using Y1H screening. The FPKM values represent the means of three biological replicates.

**Additional file 3: Table S3.** The sequences of primers used in this study.

**Additional file 4: Fig. S1.** Assays of the interaction between CmTTG1, CmMYB6 and CmbHLH2. (A) Y2H assay shows CmTTG1 interacts with CmMYB6 and CmbHLH2, and CmMYB6 interacts with CmbHLH2. (B) BiFC assay shows CmTTG1 interacts with CmMYB6 and CmbHLH2, and CmMYB6 interacts with CmbHLH2. Bars = 20 μm. (C) Yeast three-hybrid assay shows CmTTG1 doesn’t affect the interaction of CmbHLH2 and CmMYB6.

**Additional file 5: Fig. S2.** The relative expression of *CmMYB#7* in ‘Arctic Queen’ at different post-flowering stages.

**Additional file 6: Fig. S3.** Yeast assays of the interaction between CmNAC25 and promoters of five structural genes, and the interaction between CmNAC25 and CmMYB6 or CmbHLH2. (A) Y1H assay shows CmNAC25 does not bind to promoters of *CmCHS*, *CmCHI*, *CmF3H*, *CmANS*, *CmUFGT*. (B) Y2H assay shows CmNAC25 does not interact with CmMYB6 or CmbHLH2. (C) BiFC assay shows CmNAC25 does not interact with CmMYB6 or CmbHLH2. Bars = 20 μm.

**Additional file 7: Fig. S4.** EMSA assay showing that CmNAC25 directly binds to *CmDFR* promoter at the ACGT element located at -839 ~ -835 bp. * means non-specific binding band.

**Additional file 8: Fig. S5.** The floral coloration and anthocyanin accumulation of OX-CmNAC25 50# transgenic line did not change. (A) The phenotypes of CmNAC25-overexpressing lines 50# and WT plants. (B) Total anthocyanins (TA) in petals of WT and transgenic lines 50#.

**Additional file 9: Fig. S6.** DEG analysis in the transcriptome of petals of ‘Jinba’ and RNAi-*CmNAC25* 23# transgenic line at the initially senescent stage. (A) The number of upregulated and downregulated DEGs in WT vs. RNAi23#. (B) Expression pattern of structural genes in the anthocyanin biosynthesis pathway of chrysanthemum. (C) Differential expression of genes encoding MYB-like transcription factors and bHLH transcription factors. Heat maps depict normalized gene expression values (log10[FPKM + 1]), of which FPKM values represent the means of three biological replicates.

**Additional file 10. **Individual data values.

## Data Availability

All data generated or analyzed during this study are included in this published article, its supplementary information files and publicly available repositories. Transcriptome sequencing data of chrysanthemum in this study can be found at the National Center for Biotechnology Information (https://www.ncbi.nlm.nih.gov/bioproject/) with the BioProject ID PRJNA943196 (‘Arctic Queen’) and PRJNA943082 (‘Jinba’ WT and RNAi-*CmNAC25* transgenic line 23#). The individual data values for Figs. 1, 3, 5, 6, 7, and 8, as well as Additional file 5: Fig. S2, Additional file 8: Fig. S5, and Additional file 9: Fig. S6, are provided in Additional file 10.

## Abbreviations

3MaT: Anthocyanin 3-O-glucoside-6''-O-malonyltransferase
BiFC: Bimolecular fluorescence complementation
CDS: Coding sequence
GFP: Green fluorescent protein
HPLC: High-performance liquid chromatography
N.D.: No detection
OE: Over expression
ORF: Open reading frame
qRT-PCR: Quantitative real-time polymerase chain reaction
RFP: Red fluorescent protein
RNAi: RNA interference
