# Supplementary figures and images for: CmNAC25 targets CmMYB6 to positively regulate anthocyanin biosynthesis during the post-flowering stage in chrysanthemum
Source: BMC Biol. 2023 Oct 9;21:211. doi: 10.1186/s12915-023-01719-7 (PMC10561465; doi:10.1186/s12915-023-01719-7)

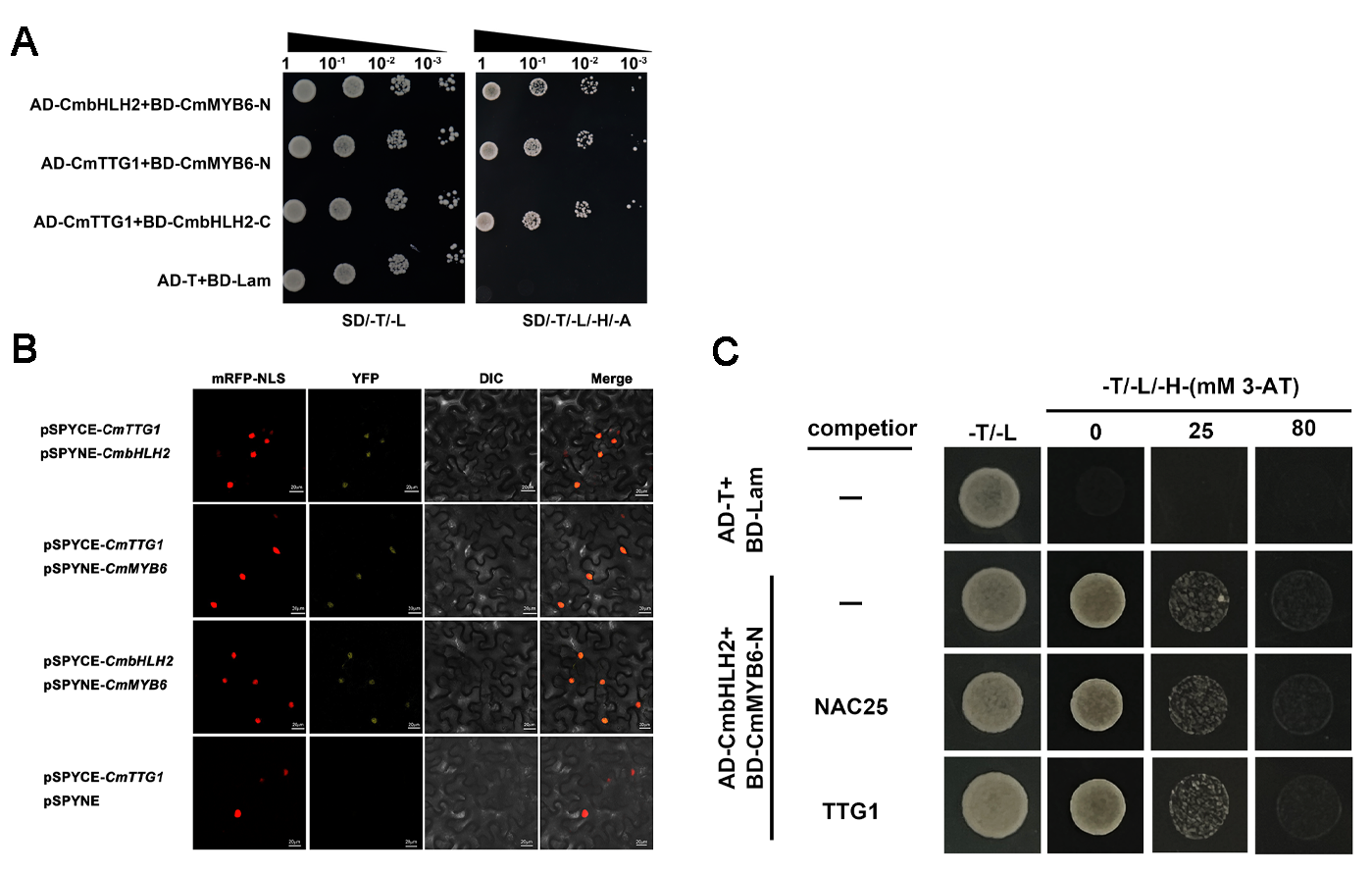

Supplement: Supplementary file 4 — Additional file 4: Fig. S1. Assays of the interaction between CmTTG1, CmMYB6 and CmbHLH2. (A) Y2H assay shows CmTTG1 interacts with CmMYB6 and CmbHLH2, and CmMYB6 interacts with CmbHLH2. (B) BiFC assay shows CmTTG1 interacts with CmMYB6 and CmbHLH2, and CmMYB6 interacts with CmbHLH2. Bars = 20 μm. (C) Yeast three-hybrid assay shows CmTTG1 doesn’t affect the interaction of CmbHLH2 and CmMYB6. [file 12915_2023_1719_MOESM4_ESM.tif]

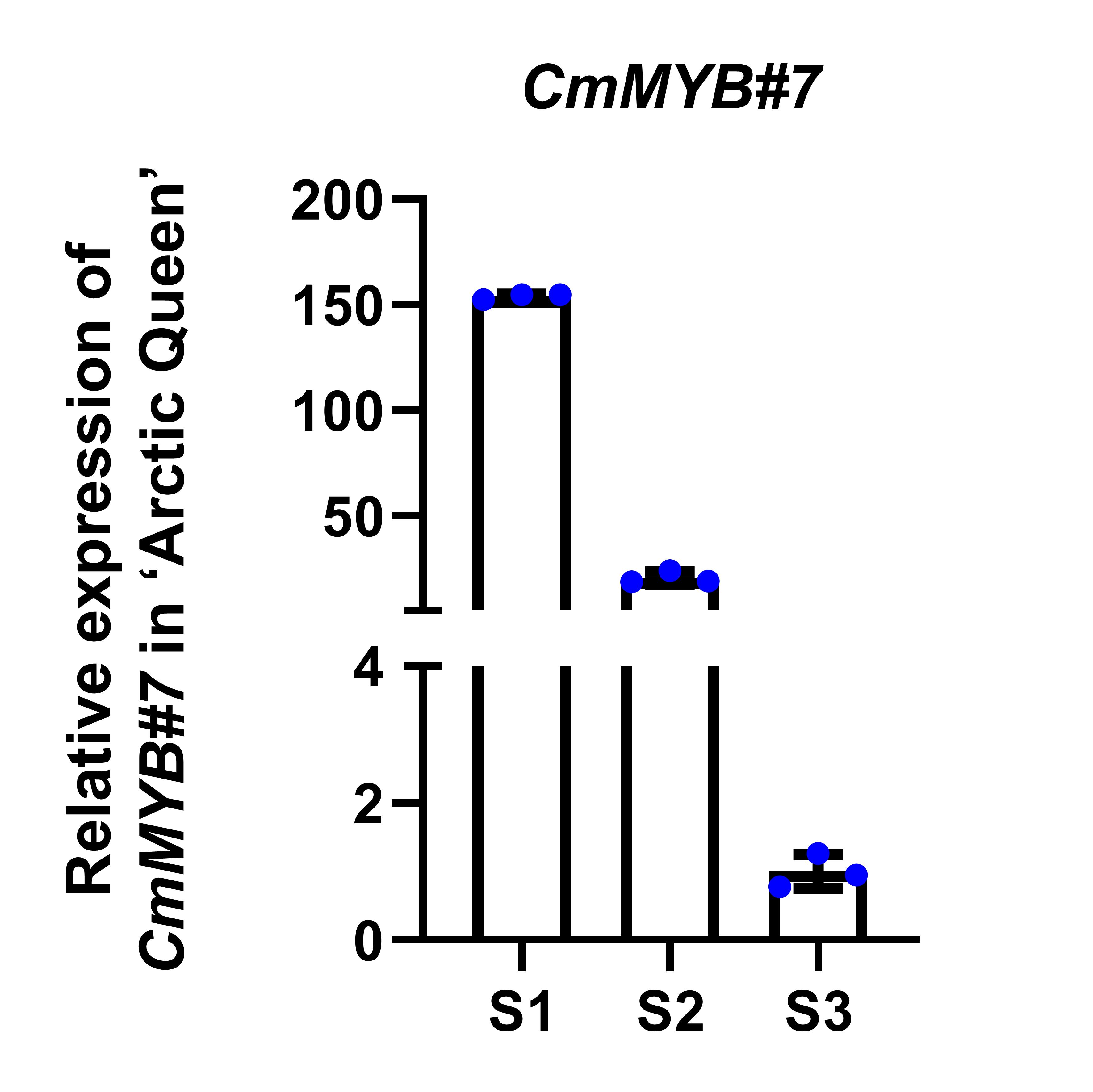

Supplement: Supplementary file 5 — Additional file 5: Fig. S2. The relative expression of CmMYB#7 in ‘Arctic Queen’ at different post-flowering stages. [file 12915_2023_1719_MOESM5_ESM.tif]

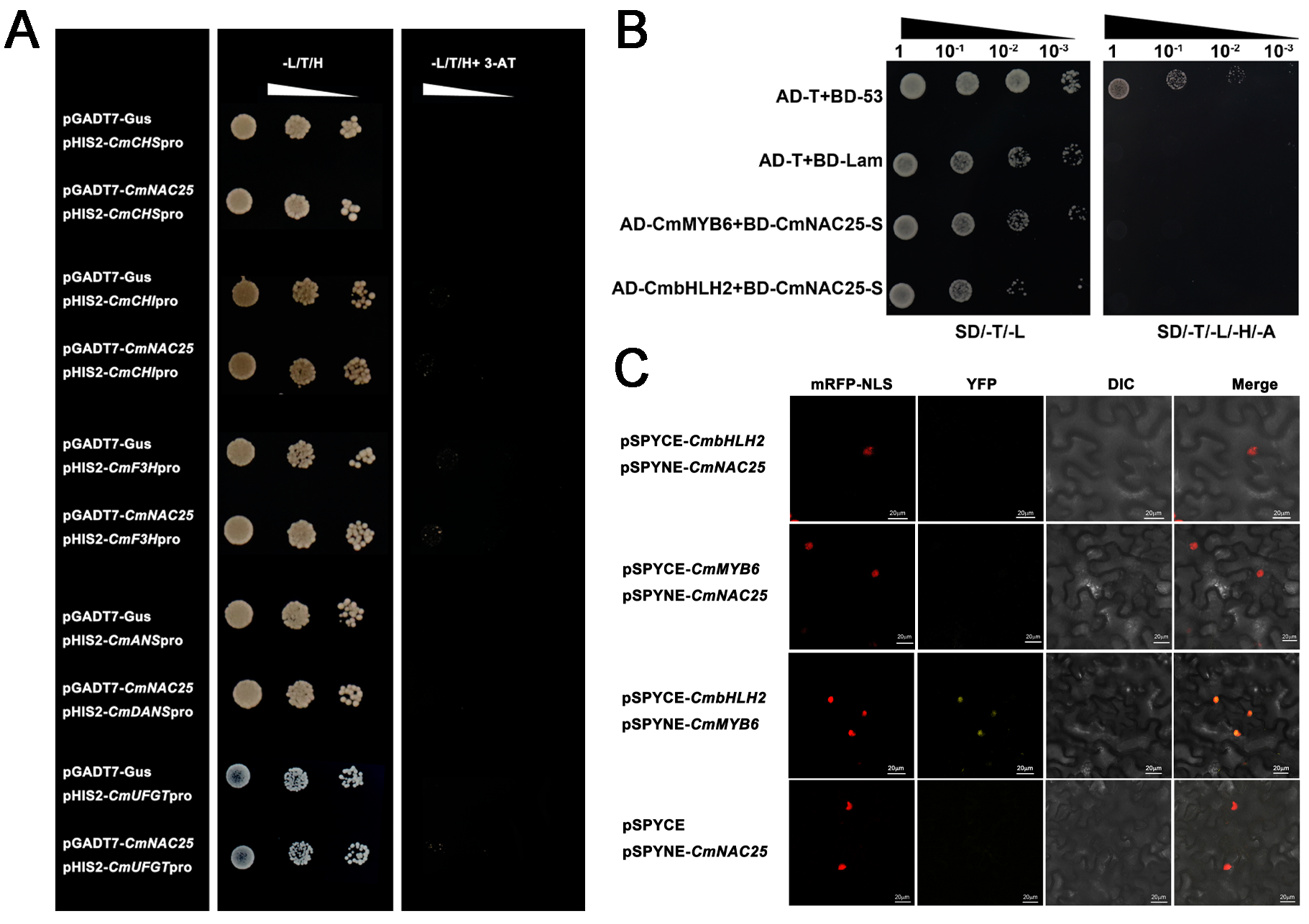

Supplement: Supplementary file 6 — Additional file 6: Fig. S3. Yeast assays of the interaction between CmNAC25 and promoters of five structural genes, and the interaction between CmNAC25 and CmMYB6 or CmbHLH2. (A) Y1H assay shows CmNAC25 does not bind to promoters of CmCHS, CmCHI, CmF3H, CmANS, CmUFGT. (B) Y2H assay shows CmNAC25 does not interact with CmMYB6 or CmbHLH2. (C) BiFC assay shows CmNAC25 does not interact with CmMYB6 or CmbHLH2. Bars = 20 μm. [file 12915_2023_1719_MOESM6_ESM.tif]

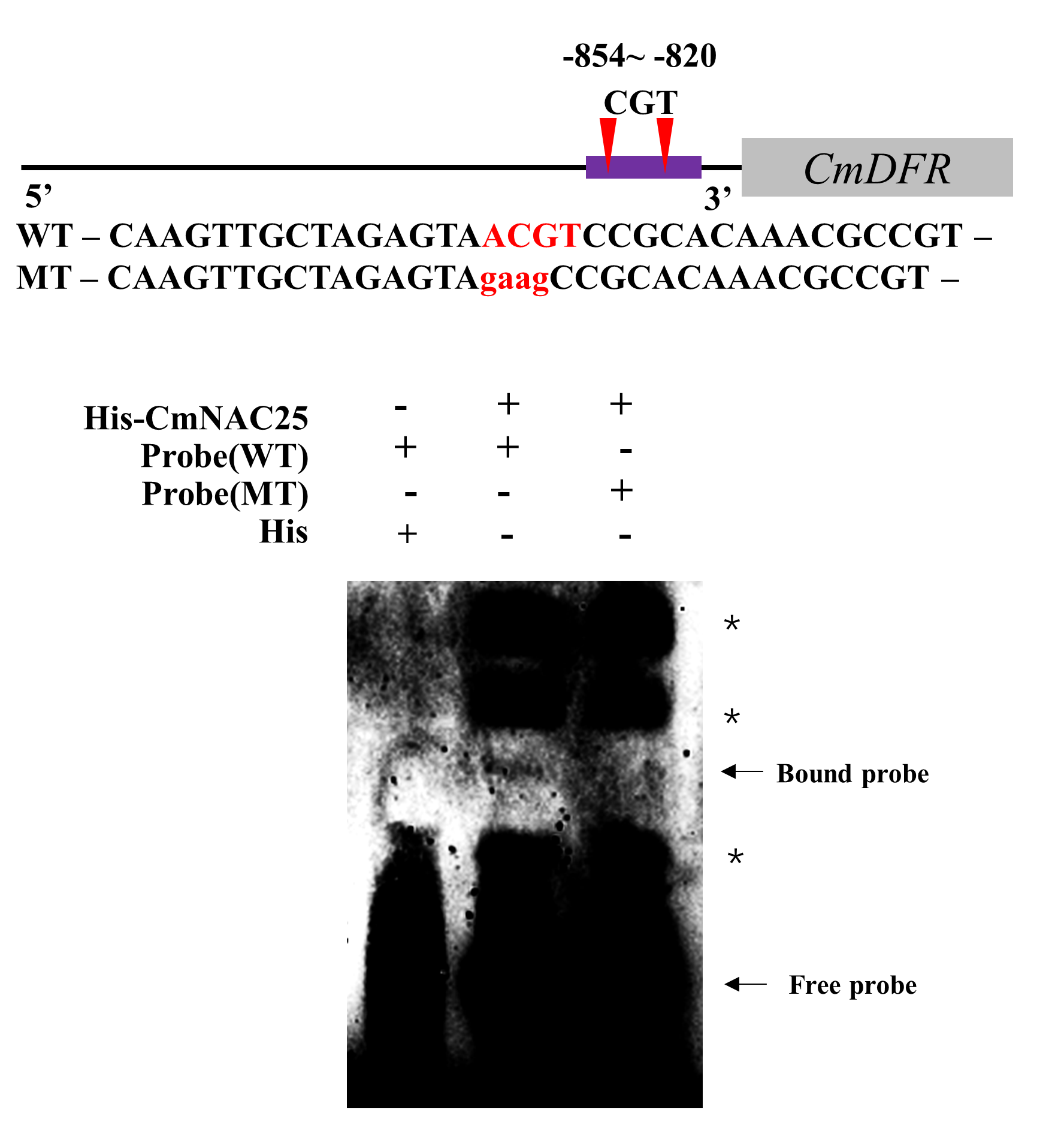

Supplement: Supplementary file 7 — Additional file 7: Fig. S4. EMSA assay showing that CmNAC25 directly binds to CmDFR promoter at the ACGT element located at -839 ~ -835 bp. * means non-specific binding band. [file 12915_2023_1719_MOESM7_ESM.tif]

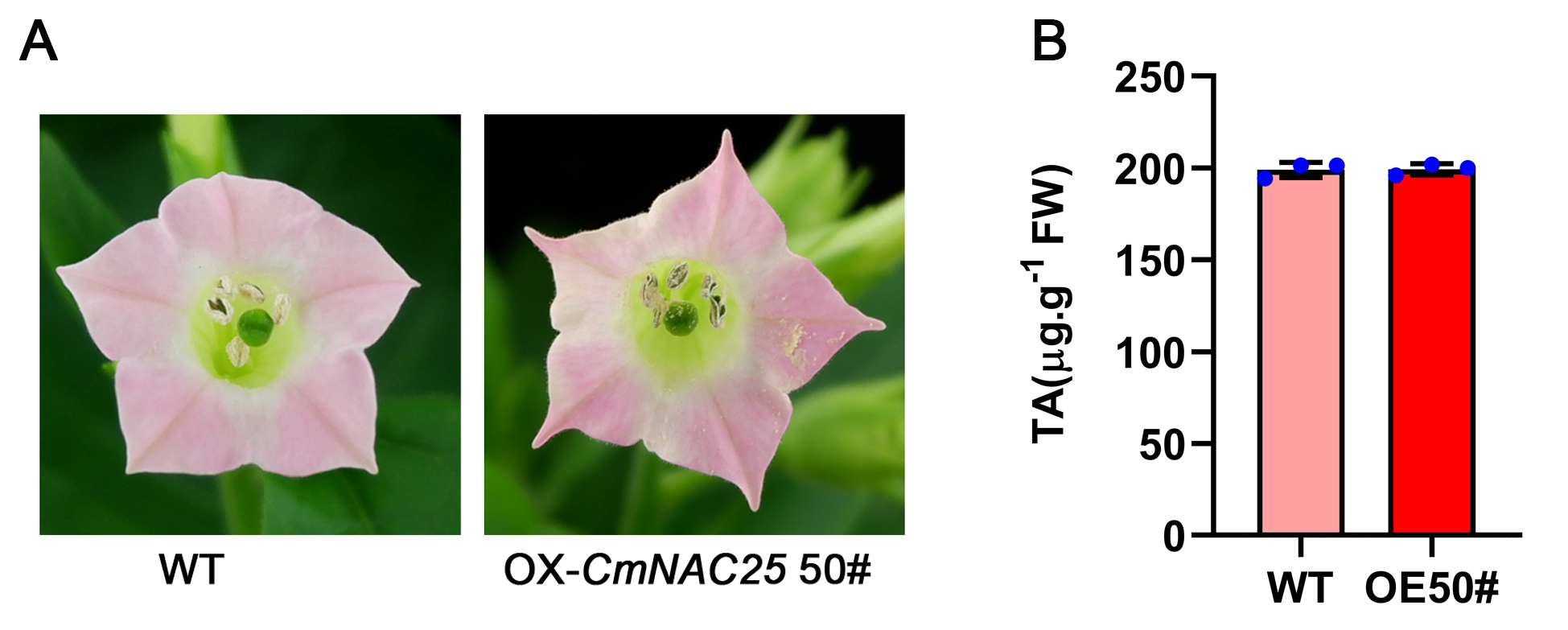

Supplement: Supplementary file 8 — Additional file 8: Fig. S5. The floral coloration and anthocyanin accumulation of OX-CmNAC25 50# transgenic line did not change. (A) The phenotypes of CmNAC25-overexpressing lines 50# and WT plants. (B) Total anthocyanins (TA) in petals of WT and transgenic lines 50#. [file 12915_2023_1719_MOESM8_ESM.tif]

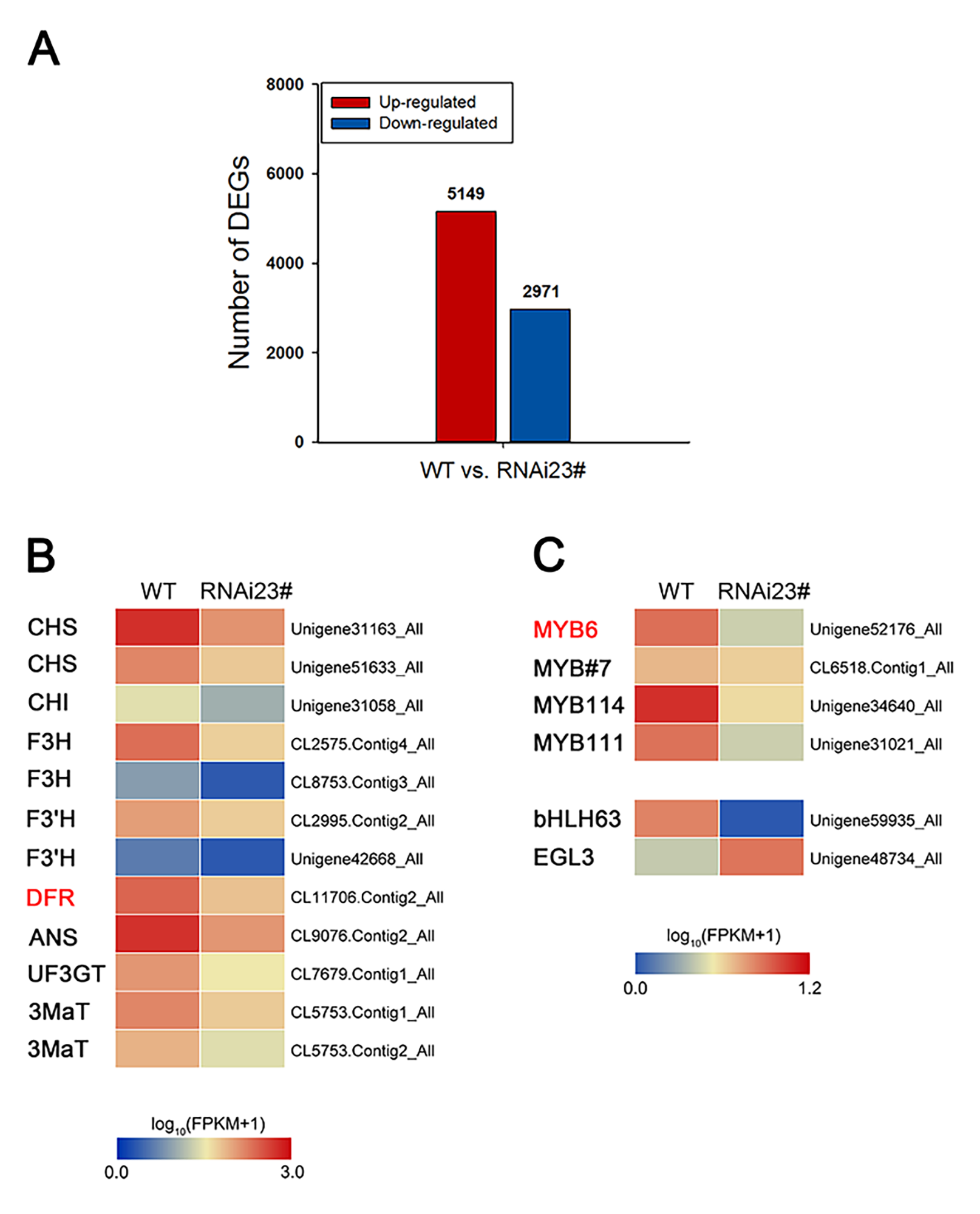

Supplement: Supplementary file 9 — Additional file 9: Fig. S6. DEG analysis in the transcriptome of petals of ‘Jinba’ and RNAi-CmNAC25 23# transgenic line at the initially senescent stage. (A) The number of upregulated and downregulated DEGs in WT vs. RNAi23#. (B) Expression pattern of structural genes in the anthocyanin biosynthesis pathway of chrysanthemum. (C) Differential expression of genes encoding MYB-like transcription factors and bHLH transcription factors. Heat maps depict normalized gene expression values (log10[FPKM + 1]), of which FPKM values represent the means of three biological replicates. [file 12915_2023_1719_MOESM9_ESM.tif]
